# A Dietary Supplement Containing Micronutrients, Phosphatidylserine, and Docosahexaenoic Acid Counteracts Cognitive Impairment in D-Galactose-Induced Aged Rats

**DOI:** 10.3389/fnut.2022.931734

**Published:** 2022-07-05

**Authors:** Qian Ren, Jianqin Sun, Danfeng Xu, Hua Xie, Mengyao Ye, Yanfang Zhao

**Affiliations:** ^1^Department of Clinical Nutrition, Huadong Hospital Affiliated to Fudan University, Shanghai, China; ^2^Department of Clinical Nutrition, Shanghai Jiao Tong University Affiliated Sixth People’s Hospital, Shanghai, China; ^3^Department of Endocrinology and Metabolism, Wenzhou Integrated Traditional Chinese and Western Medicine Hospital, Wenzhou, China

**Keywords:** dietary supplement, micronutrient, phosphatidylserine, docosahexaenoic acid, cognitive impairment, neuroinflammation, brain-derived neurotrophic factor

## Abstract

At present, it is a trend to use dietary supplements to prevent age-related cognitive impairment. This study aimed to investigate the effects of a dietary supplement enriched with micronutrients, phosphatidylserine, and docosahexaenoic acid on cognitive performance using a D-galactose (D-gal) induced aging rat model. Seven-month-old male Sprague-Dawley rats were randomly divided into five groups, including the control group, D-gal model group, and low-dose (2 g/kg body weight), medium-dose (6 g/kg body weight), and high-dose (10 g/kg body weight) dietary supplement intervention groups, which were investigated for 13 weeks. The dietary supplement intervention was found to improve cognitive performance in Morris water maze test, increase superoxidase dismutase activity, reduce malondialdehyde activity, decrease tumor necrosis factor-α and interleukin-6 concentrations, inhibit the activation of astrocytes, and elevate brain-derived neurotrophic factor protein and mRNA expression in the brains of D-gal-induced aged rats. This dietary supplement customized for the aged can be applied to the restoration of cognitive performance by enhancing antioxidant and anti-neuroinflammatory abilities, up-regulating neurotrophic factors, and inhibiting the activation of astrocytes. These results will be useful for future studies focused on implementation in humans.

## Introduction

Among all organs, the brain is the most vulnerable to oxidative stress owing to its high metabolic activity and lipid content, as well as its limited antioxidant defense mechanisms during aging (1). Thus, increased oxidative stress, which can incur structural and functional alterations in the hippocampus, ultimately leads to decrements in learning and memory, decision-making speed and sensory perception (2). To the best of our knowledge, age-related cognitive impairment is one of the major causes of disability and dependency in human aging (3).

Several human studies (4–8) and fundamental research (7, 9–11) have shown that some nutrients such as antioxidants, B vitamins, phosphatidylserine (PS), long-chain n-3 polyunsaturated fatty acids (n-3 PUFA) and polyphenols can ameliorate age-related cognitive decline and decrease the risk of dementia. Some underlying mechanisms that can potentially support the protective effects of these nutrients against brain aging are the restoration of the microstructural integrity of the hippocampus (12), prevention of glutamatergic synapse and astroglial aging (13), decreased oxidative stress and neuroinflammation (14), and increased expression of brain neurotrophins (15). Based on this situation, cognition-beneficial supplements or “nootropics” have become increasingly popular (16). However, many commercial cognition-beneficial supplements consist of agents (such as piracetam) without the approval from Food and Drug Administration (FDA) for sale (16). Furthermore, the intake levels of phytochemicals such as polyphenols have not been thoroughly investigated, and assessments of polyphenol intake levels are not fully reliable. In addition, excess or deficiency of the nutrient intake attributed to the arbitrary intake of commercial cognition-beneficial supplements—not according to one’s own nutritional status—will raise safety concerns (17). Thus, it is necessary to design dietary supplements, which can accommodate the optimal dietary intakes of the targeted age group, to avoid the risk of insufficient or excessive nutrient intake.

Due to physical changes with aging, older adults face more serious challenges in the attainment of appropriate nutrients. In order to investigate the dietary intakes of the elderly, we conducted a survey among institutional 600 older adults (aged 65 or above) in China, and found that calcium, iron, zinc, selenium, vitamin B_1_, vitamin B_2_, and vitamin C intakes of them were all lower than Chinese dietary reference intakes (DRIs) (18, 19). Therefore, a dietary supplement, mainly focused on the essential nutrients, was designed to (1) compensate for the insufficient dietary intake of micronutrients among older adults in China to reach the Chinese DRIs; and (2) improve cognitive function. The composition of the dietary supplement includes 11 vitamins, six minerals, docosahexaenoic acid (DHA), and PS as per the suggestions from experts of the Chinese Nutrition Society.

Chronic administration of D-galactose (D-gal) for 6–10 weeks to induce brain senescence and mimic the effects of natural aging has been a widely accepted model for studying the effects of therapeutic interventions on brain aging (20–23). Therefore, in the present study, we examined the effects of the dietary supplement on age-related cognitive impairment using a D-gal-induced aged rat model. The Morris water maze (MWM) test was conducted to evaluate cognitive performance. Histopathological changes in the brain were analyzed using hematoxylin-eosin (H&E) and Nissl staining. Immunofluorescence analysis was used to assess the activation of astrocytes. The hippocampus and serum concentrations and activities of superoxidase dismutase (SOD) and malondialdehyde (MDA), and serum concentrations of tumor necrosis factor-α (TNF-α) and interleukin-6 (IL-6) were measured. The expression of brain-derived neurotrophic factor (BDNF) in the hippocampus was assessed using western blot and RT-PCR. The results of our study will provide information for future clinical studies regarding this intervention.

## Materials and Methods

### Chemicals

D-gal was purchased from Sigma Aldrich (St Louis, MO, United States). ELISA kits (Rat TNF-α ELISA kit, Rat IL-6 ELISA kit, ELK Biotechnology, Wuhan, China) were used to detect TNF-α and IL-6 levels. Anti-BDNFs (ab108319), anti-GAPDH (ab37168), and anti-glial fibrillary acidic protein (GFAP) (ab33922) were purchased from Abcam (MA, United States). HRP goat anti-rabbit antibody (AS1107) was purchased from Aspen Biotechnology (Wuhan, China).

### Animals

Sprague-Dawley male rats (6 weeks old, SPF grade, weight: 229–295 g) were obtained from Shanghai SIPPR-BK Laboratory Animal Co., Ltd. (SCXK 2013-0016). The animals were housed in ventilated cages (four rats per cage) kept on a 12 h light/12 h dark cycle (light on from 08:00 to 20:00) at 23^°^C ± 2^°^C, with humidity of 50 ± 10%. Food and distilled water were provided *ad libitum*.

### Dietary Supplement and Diets

The dietary supplement was manufactured by Richen Nutritional Co., Ltd. (Shanghai, China). The compositions of the dietary supplement were determined by the suggestions, based on the dietary survey of 600 older adults (aged 65 or above) in China, by experts at the Chinese Nutrition Society (Table 1) (18, 19). It was designed to improve cognitive function while compensating for the insufficient dietary intake of micronutrients, by providing older adults with at least one sachet (per 20 g) of dietary supplements per day. The fatty acid of this dietary supplement (per 20 g) is composed of 100 mg algal-oil-derived DHA and 100 mg soybeans-derived PS. The commercial basal chow diet (AIN-93M) and the experimental diets were obtained from TROPHIC Animal Feed High-tech Co., Ltd. (Nantong, China) (24). The intervention dose was converted from the human oral dose using the human-rat species coefficient issued by the American FDA as follows (25): 20 g (human oral dose) ÷ 60 kg (standard weight of human adult) × 6.2 (the human-rat species coefficient) × 360 g (standard weight of adult rats) = 0.74 g. Based on the average intake of an adult rat per day (approximate 35 g of feed), 21 g of the dietary supplement was added to 1,000 g of feed. According to the method of function evaluation issued by the China FDA, three intervention groups should be set up in animal experiments with five times the recommended dose in human body (for rats) as one of the groups (26). Therefore, we set up low-, medium-, and high-dose groups of rats that were administered dietary supplements that were 1, 3, and 5 times the recommended human dose, respectively. To prepare the low-, medium-, and high-dose experimental diets, 1,000 g of the AIN-93M diet were mixed with approximately 21, 63, and 105 g of the dietary supplement, respectively. These doses correspond to 1 sachet (20 g), 3 sachets (60 g), or 5 sachets (100 g) of dietary supplements taken orally per day in humans. The compositions of the background AIN-93M diet, and the final amount of low-, medium- and high-dose treatment diets are listed in Table 2. All feeds were sealed and stored in the refrigerator at −20^°^C and were used within 1 week after opening.

**TABLE 1:** Composition of the dietary supplement customized for older adults (20 g per package per day).

Nutrient (per 20 g)	Dietary supplement	Chinese DRIs for adults ≥ 65 years
Energy (kcal)	76.0	2,050/1,700^a^
Protein (g)	6.0	65/55^b^
Fat (g)	0.4	−
Carbohydrate (g)	11.2	−
Dietary fiber (g)	1.2	−
Vitamin A (μg RAE)	220.0	800/700^c^
Vitamin B_1_ (mg)	1.4	1.4/1.2^c^
Vitamin B_2_ (mg)	1.4	1.4/1.2^c^
Vitamin B_6_ (mg)	1.1	1.6
Vitamin B_12_ (μg)	1.6	2.4
Vitamin C (mg)	200.0	200^d^
Vitamin D_3_ (IU)	800.0^e^	600
Vitamin E (mg α-TE)	10.0	14
Nicotinamide (mg)	11.0	13
Folate (μg)	264.0	400
Pantothenic acid (mg)	6.0	5
Sodium (mg)	60.0	2,200
Calcium (mg)	500.0	1,000
Magnesium (mg)	150.0	350
Iron (mg)	12.0	15
Zinc (mg)	7.0	11.5
Selenium (μg)	26.0	50
DHA (mg)	100.0	−
PS (mg)	100.0	−

*DHA, docosahexaenoic acid; PS, phosphatidylserine. ^a^The recommended energy intakes for male/female older adults aged 65 years or more with light physical activity. ^b^The recommended protein intakes for male/female older adults aged 65 years or more with light physical activity. ^c^The recommended nutrients intakes for male/female older adults aged 65 years or more. ^d^The proposed intake for preventing non-communicable chronic disease of vitamin C. ^e^The dose was lower than the tolerable upper intake level of vitamin D (2000 IU).*

**TABLE 2:** Components of the AIN-93M diet, and low-, medium-, and high-dose treatment diets (per kg).

Nutrient (per kg)	AIN-93M	Low-dose diet	Medium-dose diet	High-dose diet
Energy (kcal)	3,601	3,605	3,613	3,620
Protein (g)	126	129	136	142
Fat (g)	40	40	39	38
Carbohydrate (g)	727	724	717	711
Dietary fiber (g)	−	1	4	6
Vitamin A (μg RAE)	1,200	1,402	1,781	2,131
Vitamin B_1_ (mg)	5	6	9	11
Vitamin B_2_ (mg)	6	7	10	12
Vitamin B_6_ (mg)	6	7	9	11
Vitamin B_12_ (μg)	25	26	28	30
Vitamin C (mg)	−	206	593	950
Vitamin D_3_ (IU)	1,000	1,802	3,311	4,706
Vitamin E (mg α-TE)	50	59	77	93
Nicotinamide (mg)	30	41	61	79
Folate (μg)	2,000	2,230	2,664	3,064
Pantothenic acid (mg)	15	21	32	42
Sodium (mg)	1,033	1,073	1,150	1,220
Calcium (mg)	5,000	5,411	6,185	6,900
Magnesium (mg)	511	655	925	1,175
Iron (mg)	45	56	78	98
Zinc (mg)	35	41	54	65
Selenium (μg)	170	193	237	277
DHA (mg)	−	103	296	475
PS (mg)	−	103	296	475

*The fatty acid of this dietary supplement (per 20 g) is composed of 100 mg algal-oil-derived DHA and 100 mg soybeans-derived PS. DHA, docosahexaenoic acid; PS, phosphatidylserine.*

### Experimental Design

60 Sprague-Dawley male rats (6 weeks old, SPF grade, weight: 229–295 g) were randomly divided into five groups using a randomized block design according to body weight (*n* = 12 for each group). After 1 week of adaptation, the rats in the control group were fed the AIN-93M diet and treated with 0.9% saline (subcutaneous injection, s.c.). The rats in the D-gal model group (D-gal) were fed the AIN-93M diet with D-gal (300 mg/kg/day, s.c.). The rats in the low-, medium-, and high-doses of the intervention groups were treated with D-gal (300 mg/kg/day, s.c.) and were simultaneously fed with the low, medium, and high doses (2, 6, and 10 g/kg body weight dietary supplement, respectively) of the experimental diets. Since the energy differences in the AIN-93M diet, and low-, medium-, and high-dose experimental diets were subtle, the treatment diets were considered to be isocaloric.

**FIGURE 1:**
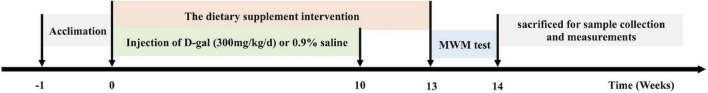
The experimental protocol of the study. After 1 week of adaptation (week –1 ∼ 0), animal modeling (week 1∼10) and the dietary supplement intervention (week 1 ∼ 13) were introduced simultaneously. After 12 weeks of supplementation, we performed the Morris water maze test (week 13), including 5 days of place navigation test and 1 day of spatial probe test. After the Morris water maze test, the rats were fasted overnight and sacrificed for subsequent tests.

The brain aging model requires D-gal injections of 50–500 mg/kg body weight for 6–10 weeks (21). Therefore, we chose chronic administration of D-gal at a dose of 300 mg/kg/day, s.c. for 10 weeks. Previous studies have shown that supplementation with micronutrients for 8–16 weeks can improve the cognitive performance of aged rats (27, 28). At first, the duration of the dietary intervention was preset as 12 weeks. However, the MWM test lasted for 1 week in which rats were still fed with the corresponding intervention diets. Therefore, the dietary intervention, in reality, lasted for 13 weeks. The experimental protocol is shown in Figure 1. The institutional and national guidelines for the care and use of animals were followed and all experimental procedures involving animals were approved by the animal ethics committee of Fudan University (approval number 201810002Z). All surgeries were performed under anesthesia and efforts were made to minimize any suffering.

### Morris Water Maze Test

Spatial learning and memory were determined using the MWM test (29, 30). Following 1 day of pre-test, the MWM test, starting in the 13th week, lasted for 6 days (including 5 days of place navigation test and 1 day of spatial probe test) (Figure 1). Besides, in order to minimize the effect of the circadian cycle, the test was performed between 8:00 and 16:00 (10). Rats were trained to use a variety of visual cues located on the pool wall to find a hidden submerged transparent platform in a circular pool (diameter 120 cm, height 45 cm). The inside of the pool was black and filled with 22^°^C ± 1^°^C water to achieve a depth of 30 cm. The pool was conceptually divided into four equal quadrants by imaging lines. During the navigation training, the invisible platform was located in the center of the fourth quadrant and remained fixed for each trial. The training was initiated by placing the rats in the water maze, from different randomly chosen start positions located in the four quadrants, in such a way that they all faced the wall of the pool. The sequence of the starting positions was constant for all rats on the first day but was changed on the training days. Each rat underwent four trials every day with a 3-min inter-trial interval. If the rat did not find the platform in the allotted time (within 90 s), it was manually guided toward it. Upon finding the hidden platform, the rat was allowed to rest on it for 10 s. After each trial, the rats were dried and kept in a dry plastic cage filled with towels to dry off. Experimental data such as time taken by the rat to reach the platform, distance covered by the rat, swimming speed, time spent in each quadrant, and the number of times a rat crossed the position of the platform were recorded. On the sixth day, the platform was removed to perform the probe trial. The rats were placed such that they were facing the wall of the tank from the second quadrant. During the test, the path length and time spent in the target quadrant, as well as the number of times a rat crossed the platform location were recorded. The rats were maintained on their respective diets throughout the duration of this test. A video camera was installed on the ceiling of the pool (placed at the center) and was attached to a video tracking system (SuperMaze software, Shanghai Xinruan Information Technology Co., Ltd., China) to monitor swim paths and latencies.

### Assay of Organ Indices and Tissue Preparation

The general appearance of the rats was observed daily throughout the study and the body weight of the rats was recorded weekly. After behavioral studies, the rats were fasted overnight and anesthetized with 10% chloral hydrate (dissolved in normal saline, 4 mL/kg body weight). Serum samples were separated from inferior vena cava and were stored at −80^°^C for subsequent experiments. Next, the rats were perfused transcardially with normal saline. The heart, liver, spleen, and thymus gland were isolated, blotted dry, and weighed to calculate the organ index according to the following equation: organ index = organ weight (g)/body weight (g) × 100%.

The brains of the rats were carefully removed and washed with ice-cold normal saline and weighed separately. In each group, four brains were used for histopathological examination and immunofluorescence analysis and another eight brains were used for biochemical analysis. Brains were sagittally cut into two halves and paraffin-embedded. The left hemispheres were post-fixed in 4% paraformaldehyde for histopathological examination and immunofluorescence analysis. The hippocampi were immediately separated from the right hemispheres on a cold plate, collected in 0.5 mL EP tubes, and stored at −80^°^C for biochemical analysis, western blotting, and mRNA expression studies.

### H&E and Nissl Staining

H&E and Nissl staining were performed (31) for histological examination and evaluation of neuronal loss.

### Immunofluorescence Analysis

Paraffin sections were prepared, dewaxed, and rinsed thrice with distilled water. Citrate buffer (pH 6.0) was used for heat-induced antigen retrieval. The sections were treated with 3% bovine serum albumin (BSA) for 30 min to eliminate non-specific binding. The sections were then incubated with a primary antibody (anti-GFAP, 1:300) at 4^°^C in a light-proof wet box overnight, followed by incubation with a secondary antibody (HRP goat anti-rabbit antibody, 1:400) at room temperature (25–30^°^C) for 50 min. The sections were washed three times with phosphate-buffered saline (PBS) (pH 7.4) on a decolorizing shaking bed and each washing step was performed for 5 min. After the sections were slightly dried, DAPI dye solution was dripped into the circle and incubated at room temperature for 10 min. Brain sections were washed and visualized with anti-fluorescence quenching (Record Biotechnology Co., Ltd, Shanghai, China). The sections were observed under an inverted fluorescence microscope (Nikon, Tokyo, Japan) (ultraviolet excitation wavelength 330–380 nm, emission wavelength 420 nm, FITC green excitation wavelength 465–495 nm, emission wavelength 515–555 nm, CY3 red excitation wavelength 510–560 nm, emission wavelength 590 nm, 200× magnification) and the images were collected. The screenshots from the dentate gyrus (DG) region (200× magnification) were captured in four slides for each group. The staining of the GFAP-positive cells was analyzed using computerized planimetry Image-pro plus 6.0 (Media Cybernetics, Inc., Rockville, MD, United States). The results of four separate measurements for the DG section are expressed as mean ± standard error.

### Determination of Antioxidant Activity

The hippocampi of the rats were homogenized in tissue total protein lysis buffer (1 g tissue in 9 mL lysis buffer). The homogenate was then centrifuged at 4,000 × *g* for 10 min and the supernatant was used for the subsequent tests. The concentration of protein was measured using a commercial bicinchoninic acid (BCA) protein assay kit and BSA was used as the standard. MDA levels and SOD activity in both the hippocampus homogenate and serum were assayed using commercially available kits (Nanjing Jiancheng Bioengineering Research Institute, Nanjing, China) according to the manufacturer’s instructions (32).

### Analysis of Inflammatory Cytokines in the Serum

Serum concentrations of TNF-α and IL-6 were measured using TNF-α and IL-6 ELISA kits (ELK Biotechnology, Wuhan, China) according to the manufacturer’s instructions.

### Total RNA Isolation and Real-Time RT-PCR

Total RNA was isolated from the hippocampus using Trizol reagent (#15596-026, Ambion, Austin, TX, United States) according to the manufacturer’s instructions. The RNA integrity was confirmed by A260/280 ratio and gel electrophoresis (Beijing Junyi Electrophoresis Co., Ltd.) analysis, cDNA was synthesized from 3.52 μg RNA sample, 2 μL Oligo (dT) 18 (10 μM), 4 μL dNTP (2.5 mM), 4 μL 5XHiscript Buffer, 0.5 μL ribonuclease inhibitor, up to 20 μL RNase free ddH_2_O at 25^°^C for 5 min, 50^°^C for 15 min, 85^°^C for 5 min followed by 4^°^C for 10 min by HiScript Reverse Transcriptase (RNase H) (Vazyme Biotech Co., Ltd., Nanjing, China). The cDNA was diluted 5 times and 4 μL cDNA, 0.4 μL forward primer (10 μM), 0.4 μL reverse primer (10 μM), 10 μL SYBR Green qPCR Master Mix (Vazyme Biotech Co., Ltd., Nanjing, China), 0.4 μL 50X ROX Reference Dye 2, and 4.8 μL H_2_O were used for quantitative RT-PCR that was performed with gene-specific primers on a QuantStudio™ 6 Flex real-time thermocycler (Applied Biosystems, Forster City, CA, United States). Rat-specific primers were designed and synthesized by TSING KE Biological Technology (Beijing, China). Primer sequences used for the GAPDH (internal control) and BDNF amplification are shown in Table 3. The following cycle conditions were used: 50^°^C for 2 min, 95^°^C for 10 min, followed by 40 cycles of 95^°^C for 30 s, and 60^°^C for 30 s. Relative expression levels of gene expression were calculated and expressed as 2^–Δ^ ^Δ^ *^Ct^*. Data are expressed as the relative level of the target gene expression in the young control group normalized to that of GAPDH. Each experiment was performed in triplicate with eight independent samples per group.

**TABLE 3:** Primer sequences of RT-PCR analysis.

Gene name	Primer sequence	Size
Rat *BDNF*	Forward: 5′-ACAGCAACAGGGTGGTGGAC-3′ Reverse: 5′-TTTGAGGGTGCAGCGAACTT-3′	253 bp
Rat *GAPDH*	Forward: 5′-ATCCGAGAGCTTTGTGTGGA-3′ Reverse: 5′-GTGCTCAAAAGTGTCAGCCA-3′	249 bp

*BDNF, brain-derived neurotrophic factor.*

### Western Blot Analysis

The hippocampi were rinsed 2–3 times with pre-cooled PBS to remove any blood and were completely homogenized with a homogenizer in ice-cold radio-immunoprecipitation assay lysis buffer (Aspen Biotechnology, Wuhan, China). Protease inhibitor cocktail (Aspen Biotechnology) was added to the hippocampi minutes before homogenization. The homogenate was transferred to a centrifuge tube for oscillation and was then left in an ice bath for 30 min. The homogenate was centrifuged at 13,000 × *g* for 5 min (4^°^C) and the supernatant was collected for subsequent analysis. The protein content of the supernatant was estimated using a BCA protein assay kit (Aspen Biotechnology).

Samples were mixed with 5X protein buffer (Aspen Biotechnology) and incubated for 5 min at 98^°^C before loading. Equal amounts of protein (40 μg) were separated using 12% sodium dodecyl sulfate-polyacrylamide gel electrophoresis and were then transferred to polyvinylidene difluoride membranes (Merck Millipore, Billerica, MA, United States). After blocking with 5% (w/v) skim milk for 1 h, the membranes were incubated with the primary antibodies [BDNF (1:1,000) and GAPDH (1:10,000)] overnight at 4^°^C, followed by incubation with HPR goat anti-rabbit antibody (Aspen Biotechnology) at room temperature for 30 min. The membranes were rinsed with buffer and the proteins were detected using an ECL detection reagent (Aspen Biotechnology) according to the manufacturer’s instructions. The X-ray films were scanned and quantitative analysis of each band area and density in blots was performed using AlphaEaseFC software (Alpha Innotech, CA, United States).

### Statistical Analyses

Statistical analyses were performed with SPSS R24.0.0.0 (IBM Corporation, NY, United States) and Prism 7 for Mac OS X Version 7.0a (GraphPad Software Inc., San Diego, CA, United States). A *p*-value < 0.05 was considered statistically significant and the results are expressed as the mean ± standard error of the mean (SEM). The primary outcome was the time spent in the target quadrant, as well as the number of times a rat crossed the platform location in the MWM test. The secondary outcomes included the immunofluorescence analysis, the hippocampus and serum concentrations and activities of SOD and MDA, and serum concentrations of TNF-α and IL-6, as well as the expression of BDNF in the hippocampus. The group size was based on previous experimental protocols to allow for the detection of differences for the MWM test with a sufficient power of 80% at the level of significance of 0.05 (10). Twelve rats were included in each treatment group to obtain valid statistical data and account for unexpected mortality during the 13 weeks of treatment. The one-way ANOVA was conducted on comparisons of different groups, followed by the *post hoc* Tukey test. A repeated-measures two-way ANOVA was used for comparison of the main effects of time (day) and treatment and the time × treatment interaction in the navigation training. A trend analysis was also performed for the primary outcomes, i.e., time spent in the targeted quadrant and the number of times crossing the targeted quadrant.

## Results

### The Dietary Supplement Increased Body Weight and Organ Indices of the D-Galactose Induced Aged Rats

**FIGURE 2:**
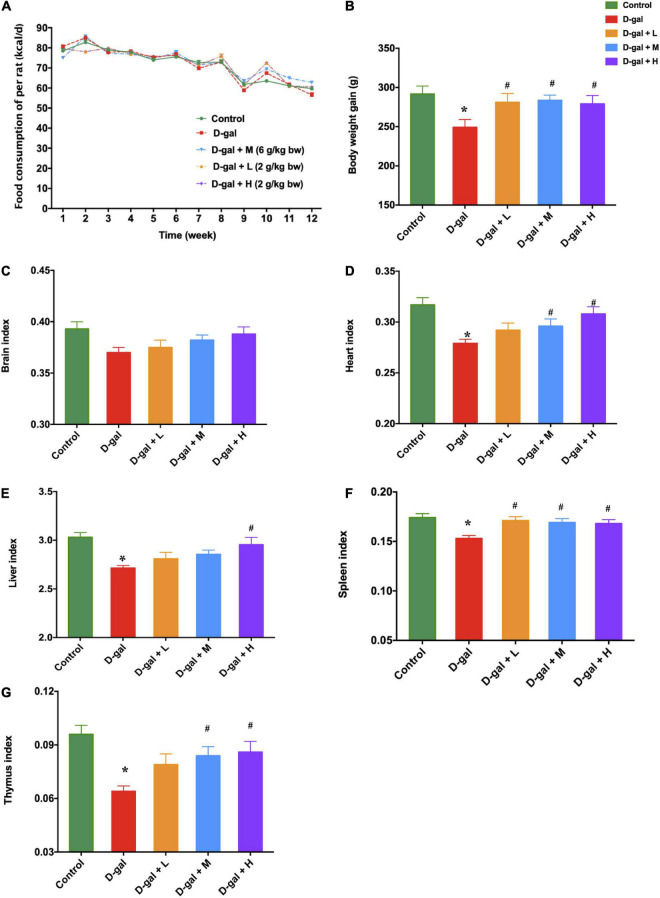
Effects of the dietary supplement on mean energy of food consumption **(A)**, mean body weight gain **(B)**, and organ indices **(C–G)** in D-gal induced aged rats at the end of supplementation (week 13). The data are presented as mean ± SEM (*n* = 12). *p < 0.05 when compared with the control group; ^#^*p* < 0.05 when compared with the D-gal group. D-gal, D-galactose; SEM, standard error of mean.

After 10 weeks of D-gal treatment, rats in the D-gal group showed reduced enthusiasm and physical inactivity, together with loss of hair, compared to the rats in the control group. The mean energy of food consumption of the rats in the five groups was similar each week (Figure 2A); however, the mean body weight gain of the rats in the D-gal group was significantly lower than that of the rats in the control group (*p* < 0.05) (Figure 2B). Moreover, the rats in the three dietary supplement groups showed a significant increase in their body weight compared with the rats in the D-gal group (*p* < 0.05). Meanwhile, the organ indices for all organs except the brain were significantly smaller in the D-gal group than those in the control group (Figures 2C–G) (*p* < 0.05). The brain indices of the D-gal-induced aged rats showed a declining trend (*p* = 0.095), while they were not substantially different from those of the control group. However, the heart index (Figure 2D) and thymus index (Figure 2G) of the rats in the medium- and high-dose groups were significantly increased compared with those in the D-gal group. The spleen index (Figure 2F) of the rats in three treatment groups were all significantly increased compared with those in the D-gal group. Only the liver indices in the high-dose group were significantly increased, compared with those in the D-gal group (Figure 2E) (all *p* < 0.05).

### The Dietary Supplement Improved Spatial Learning and Memory of the D-Galactose Induced Aged Rats Assessed by Morris Water Maze Test

**FIGURE 3:**
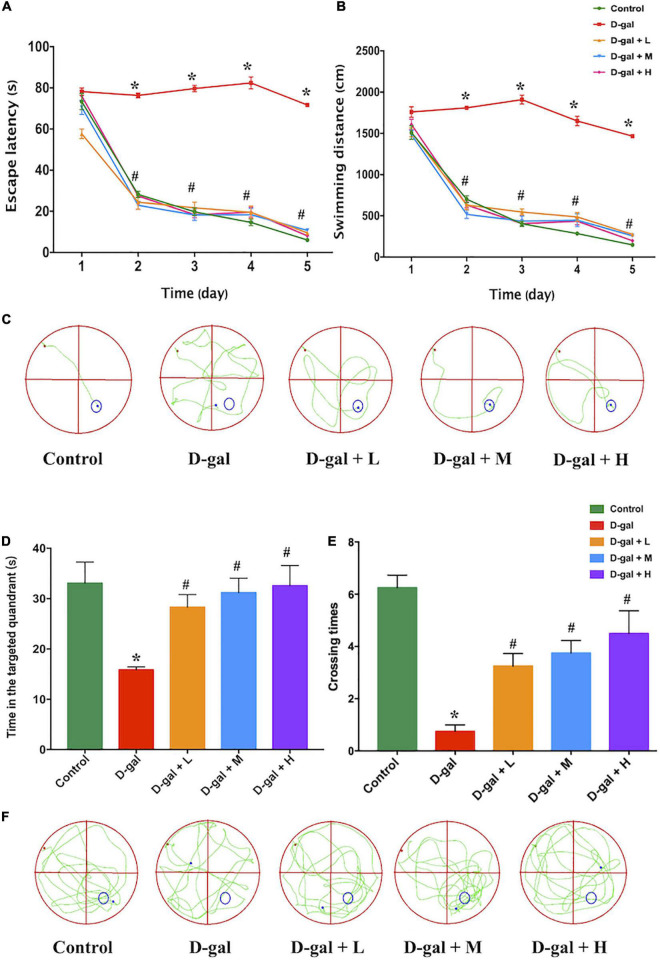
Effects of the dietary supplement on learning and memory impairment in Morris water maze test (*n* = 4 in each group). The escape latencies **(A)**, swimming distance **(B)**, the typical swimming path on the fifth day **(C)** in the navigation training, the time in the targeted quadrant **(D)**, platform crossing times **(E)**, and the typical swimming path **(F)** in the probe trial. Values are presented as means ± SEM. *p < 0.05 when compared with the control group; ^#^*p* < 0.05 when compared with the D-gal group. D-gal, D-galactose; SEM, standard error of mean.

The MWM test was used to investigate the effects of the dietary supplement on cognitive impairment in D-gal-induced aged rats. Figures 3A,B revealed D-gal group as the only group that did not show a progressive decline in either escape latency or swimming distance with training. Repeated measures two-way ANOVA for escape latencies and path to a hidden platform during MWM acquisition revealed significant main effects for day and for treatment (*F*_*treatment*_ = 69.12, *p* < 0.001; *F*_*day*_ = 478.07, *p* < 0.001; *F*_*interaction*_ = 2.15, *p* > 0.05). The results demonstrated that the spatial learning ability of the rats in the D-gal group was significantly impaired during the 5-day navigation training because their escape latency and swimming distance were longer than those of the control group (Figure 3C). However, the escape latency and swimming distance of rats in the dietary supplement intervention groups (low-, medium-, and high-dose) were significantly decreased, compared with those in the D-gal group (all *p* < 0.05) (Figures 3A,B).

In the spatial probe trial, rats in the D-gal group spent less time in the targeted quadrant (Figure 3D) and crossed the location of the removed platform fewer times (Figure 3E) than the rats in the control group (both *p* < 0.05), whereas the rats in the three dietary supplement intervention groups spent more time in the targeted quadrant and crossed the target location more times than the rats in the D-gal group (all *p* < 0.05) (Figures 3D–F). The dose-effect trends of the time spent in the targeted quadrant (*P*_*trend*_ < 0.001) and the number of times crossing the targeted quadrant (*P*_*trend*_ < 0.001) among the three dietary supplement groups were statistically different.

### Histopathological and Immunopathological Changes in the Dentate Gyrus Region of Hippocampi

**FIGURE 4:**
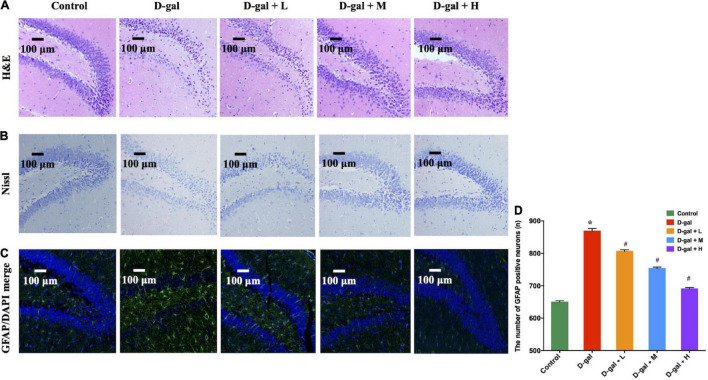
The representative photomicrographs of H&E staining **(A)**, Nissl staining **(B)** and immunofluorescence staining of GFAP (green) **(C,D)** in the DG region of the hippocampus (*n* = 4 in each group). In photomicrographs of immunofluorescence, astrocytes were positive for GFAP with ramified branches. The images of the scale bar represent 100 μm, magnification = 200X. Values are presented as means ± SEM. *p < 0.05 when compared with the control group; ^#^*p* < 0.05 when compared with the D-gal group. DG, dentate gyrus; GFAP, glial fibrillary acidic protein; SEM, standard error of mean.

The histopathological changes in the DG region of the brain were further explored using H&E (Figure 4A) and Nissl staining (Figure 4B). Neurons in the DG region of the rats in the control group showed normal morphology with well-preserved cytoplasm, prominent nucleus, and nucleolus. However, neuronal degeneration and nucleus shrinkage were observed in the neurons in the hippocampal DG regions of the rats in the D-gal group. In contrast, the neuron damage was alleviated in the dietary supplement intervention groups.

GFAP expression indicates the overaction and inflammatory response of astrocytes in hippocampal aging (33). The D-gal-treated rats showed higher GFAP expression in the DG regions, compared with the rats in the control group (Figures 4C,D). However, the expressions of GFAP in the DG region in the three intervention groups were decreased, compared with those of the rats in the D-gal group (all *p* < 0.05).

### The Dietary Supplement Decreased Malondialdehyde Concentration and Increased Superoxidase Dismutase Activity in Brain and Serum

**FIGURE 5:**
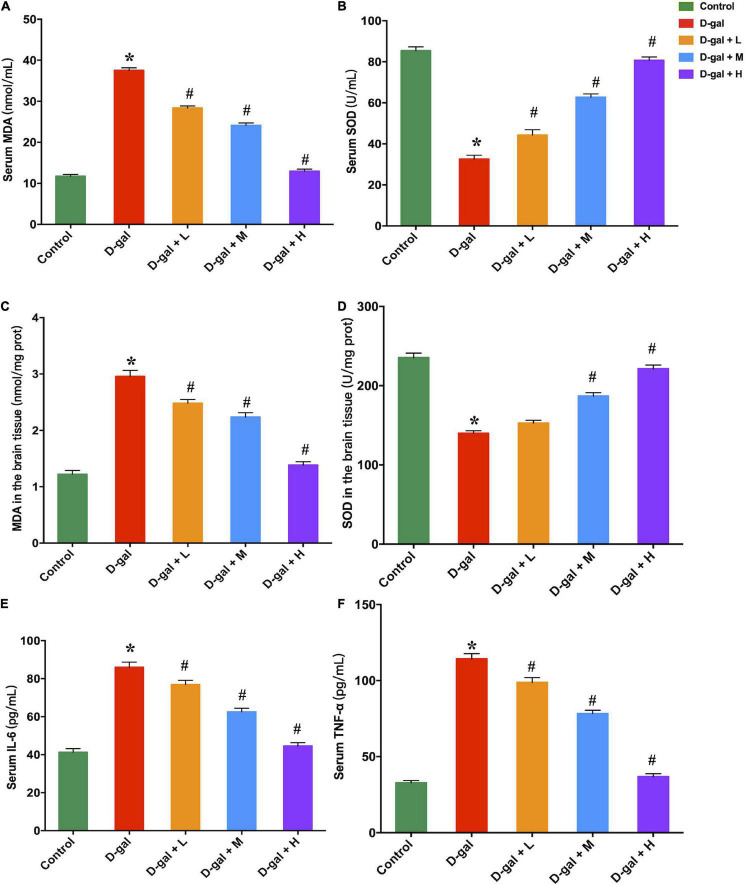
Effects of the dietary supplement on MDA **(A,C)** and SOD **(B,D)** contents in both serum and brain tissues, and serum IL-6 **(E)** and TNF-α **(F)** concentrations (*n* = 8 in each group). Values are presented as means ± SEM. *p < 0.05 when compared with the control group; ^#^*p* < 0.05 when compared with the D-gal group. IL-6, interleukin-6; MDA, malondialdehyde; SEM, standard error of mean; SOD, superoxidase dismutase; TNF-α, tumor necrosis factor-α.

MDA and SOD concentrations in the hippocampi and serum were measured to assess the effect of dietary supplement intervention on oxidative capacities. The D-gal treatment significantly decreased the SOD activities and increased the MDA concentrations (Figures 5A–D). In contrast, the low-, medium-, and high-dose dietary supplement treatments down-regulated MDA accumulation in both serum and brain, and up-regulated production of SOD in serum (Figures 5A–C). However, only medium- and high-dose dietary supplement treatments up-regulated the SOD activities in brain (Figure 5D).

### The Dietary Supplement Decreased Serum TNF-α and IL-6 Concentrations

The serum concentrations of TNF-α and IL-6 in the D-gal group were significantly higher than those of the control group (Figures 5E,F). The dietary supplement intervention (low-, medium-, and high-dose) restrained the D-gal-induced alterations in the serum levels of pro-inflammatory cytokines (all *p* < 0.05).

### The Dietary Supplement Upregulated Brain-Derived Neurotrophic Factors Expression in the Hippocampi

**FIGURE 6:**
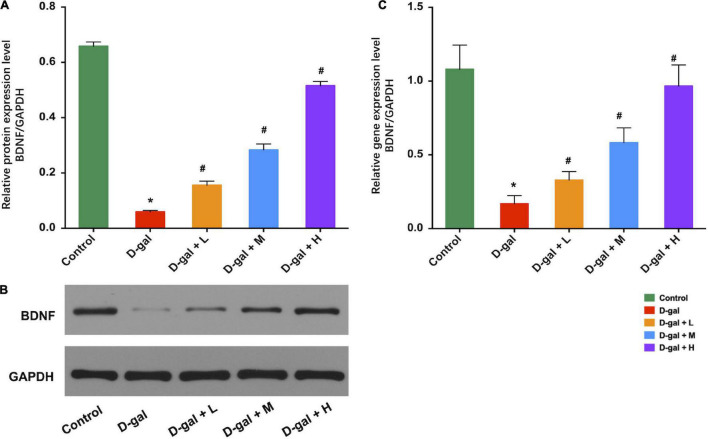
Effects of the dietary supplement on expression of BDNF in hippocampi (*n* = 8 in each group). Supplement of Dietary supplement can increase the expression of BDNF protein levels **(A,B)** and the expression of *BDNF* mRNA levels **(C)** in hippocampi. Values are presented as means ± SEM. *p < 0.05 when compared with the control group; ^#^*p* < 0.05 when compared with the D-gal group. BDNF, brain-derived neurotrophic factor; D-gal, D-galactose; SEM, standard error of mean.

Western blot analysis revealed that the hippocampal BDNF expression levels in the D-gal group were significantly decreased, compared with those of the control group (*p* < 0.05), whereas the three dietary supplement intervention group showed substantially increased BDNF expression levels compared to that of the D-gal group (all *p* < 0.05) (Figures 6A,B). *BDNF* mRNA expression in the hippocampus demonstrated a similar trend (Figure 6C).

## Discussion

The present study aimed to investigate whether the dietary supplement could improve age-related cognitive impairments using the D-gal-induced aged rat model. In the aged rats induced by D-gal, it was discovered that the dietary supplement intervention reduced learning and memory deficits, reversed changes in lower organ indices, decreased MDA levels, increased SOD activities, decreased TNF-α and IL-6 concentrations, attenuated astrocyte reactivities, and upregulated expression of BDNF.

Aging is characterized by changes in appearance, body weight, and organ indices (34). The administration of D-gal in rats can accelerate the development of these symptoms (21), manifested as significant decreases in heart, liver, thymus, and spleen indices (35, 36). In line with the results, our study also demonstrated that the administration of D-gal for 10 weeks caused atrophy of the heart, liver, spleen, and thymus. Nevertheless, administration of the dietary supplement for 13 weeks restored the organ indices of the rats in three supplement groups closer, than in the D-gal group, to those of the control group, in which the medium dose of the dietary supplement increased heart, spleen, and thymus indices, and the high dose of the dietary supplement increased heart, spleen, liver, and thymus indices.

The hippocampus plays an important role in spatial learning and memory (37). However, neurogenesis in the DG area of the hippocampus shows a continuous decline as the brain ages (38). This decline can be attributed to the age-related exhaustion of neural stem cells and progenitor cells (NSCs/NPCs) through their conversion into mature hippocampal astrocytes (39). Furthermore, the reduction in neurogenesis contributes to cognitive deficits in hippocampal learning and spatial memory (40). In the present study, the dietary supplement promoted retention of normal neurons, which manifested in the form of an increased number of normal neurons in the H&E and Nissl staining. Folate in the dietary supplement may promote neurogenesis by stimulating the proliferation of NSCs (41). Vitamin B_12_ and DHA may also contribute to the microstructural integrity of the hippocampus as they act as precursors for the formation of neuronal membranes and synapses (12, 42). Additionally, vitamins A and D may improve hippocampal neurogenesis by regulating the retinoic acid signaling pathway on the action of glucocorticoids and differentiation of NPCs (43, 44).

Chronic injection of D-gal can disrupt the oxidative/anti-oxidative balance, exert reactive oxygen species (ROS), and lead to impaired learning and memory function (45). Excessive ROS not only potently inhibits neurogenesis but also increases neuroinflammation and neurodegeneration (46). Our results showed that the dietary supplement protected the hippocampus against oxidative stress by enhancing the SOD activity (one of the important anti-oxidative enzymes) to remove ROS accumulated during aging. The decrease in the MDA level, an indication of lipid peroxidation, also contributed to the reduction of ROS. Many nutrients including vitamins C, E, and D, and iron are associated with oxidative stress; moreover, oxidative stress and inflammation pathways are interrelated (47–49). For example, the administration of vitamin E (α-tocopherol at a dose of 100 mg/kg once daily via oral gavage for 6 weeks) has been shown to prevent high-fat high-carbohydrate diet-induced memory impairment, with normalized SOD activity, thiobarbituric acid reactive substances levels, and oxidized glutathione in the hippocampus of adult male Wistar rats (50). Similarly, in male Wistar rats, vitamin D supplementation preserves high-fat induced cognitive impairment, increases glutathione peroxidase and SOD activities, and reduces MDA concentration (51). These results suggest that the dietary supplement may attenuate D-gal-induced cognitive impairment, possibly by activating antioxidant enzymes and eliminating free radicals in the hippocampus.

The presence of long-lasting increments in pro-inflammatory cytokines in the hippocampus, such as Il-6, IL-1β, and TNF-α, has led to the implication of neuroinflammation in brain aging and cognitive impairments (52). Additionally, GFAP-positive neurons, which serve as markers of activated astrocytes, have also been identified as a major brain-derived source of inflammatory cytokines (53). In our study, the serum levels of IL-6 and TNF-α were significantly lower in the dietary supplement groups than those in the D-gal group, possibly due to the anti-inflammatory activities of the nutrients in dietary supplements. In 22-month-old Wistar rats fed an n-3 deficient diet from birth, poor n-3 PUFA status exacerbates reduced glutamatergic transmission and its related astroglial regulation in the hippocampus (13). However, the amelioration of cognitive deficits came off in conjunction with the picking-up of the level of DHA in the brain in old senescence-accelerated prone 8 mice (54). Further, the administration of vitamin C (200 and 400 mg/kg body weight) can restore colchicine induced memory impairments and also decrease hippocampal TNF-α and IL-1β concentrations in colchicine induced Alzheimer’s disease rats (48). Vitamin D supplementation (1 μg/kg vitamin D daily by intragastric gavage for 10 days) also attenuates neuroinflammation through the up-regulation of the anti-inflammatory cytokines (*IL-10*, *IL-4*, and *TGF-*β) mRNA in a Parkinson’s disease animal model (55). Another study found that supplementation of DHA, eicosapentaenoic acid, uridine, choline, phospholipids, folate, vitamins B_12_, vitamin B_6_, vitamin C, vitamin E, and selenium decreases assessed neuroinflammatory response and improves the neural function and structure, which is assessed by magnetic resonance imaging and positron emission tomography, in middle cerebral artery occlusion C57BL/6J mice after ischemic stroke (56). These results suggest that the dietary supplement can counteract neuroinflammation and astrogenesis to protect the hippocampal function.

BDNF is one of the most important neurotrophic factors in the brain and primarily exists in the hippocampus to regulate learning and memory capacity (57). It plays a central role in neurogenesis, survival, growth, and synaptic plasticity (58), whose expression decreases during aging (59). The neuroprotective effects of BDNF in the regulation of neurocognitive functions, such as learning, memory, synaptic transmission, and plasticity, have been demonstrated in some studies (60). BDNF can also decrease inflammatory factors (including TNF-α, IL-1β, and IL-6), increase anti-inflammatory factors (IL-10), and inhibit hippocampal apoptosis to reduce neuroinflammation (61). The supplement of vitamin B_1_ (10 mg/kg body weight by oral gavage) every other day for 30 days has been found to increase hippocampal BDNF levels and improve spatial memory performance in a chronic stress model of Wistar rats (62). Another study showed that supplementation of a diet mixed with vitamin B_12_ and fish oil derived DHA and eicosapentaenoic acid, increases BDNF levels in both the hippocampus and cortex and improves cognitive performance in Wistar albino rats (15). Moreover, vitamin D can reverse cognitive impairments by the elevation of hippocampal BDNF concentrations in high-fat diet-induced obese Wistar rats (orally with 500 IU/kg vitamin D for 5 weeks) (63) and protect against hippocampal apoptosis in seizure Sprague Dawley rats model (orally with 500 IU/kg for 2 weeks) (64). In accordance with the above results, our results indicate that the dietary supplement increased the BDNF levels in the D-gal-induced aged rats, which is consistent with the observed improvement in cognitive function. Hence, we believe that the improvement of cognition could be partially attributed to the increase in the BDNF levels.

In this study, we designed a cognition-beneficial dietary supplement targeting Chinese older adults based on the dietary survey and investigated the effect of the dietary supplement on cognition using D-gal-induced aged rats. The results provide a theoretical basis for future clinical studies regarding this intervention. We observed that the time spent in the targeted quadrant and the number of times crossing the targeted quadrant have a dose effect among the three dietary supplement groups. In addition, as treatment dose increased, parameters including serum MDA, SOD, IL-6, TNF-α, and BDNF in hippocampi tend to, respectively, draw near their optimal values. However, even the low-dose dietary supplement can improve the cognitive deficits of the rats, which means one sachet (20 g) per day of this dietary supplement may potentially improve the cognitive decline in the elderly. In terms of population-based intervention studies, the Chinese DRIs for older adults need to be considered, the optimization of a supplement approach in humans, maybe at a dose of 1–2 sachets/day, requires further clinical trials.

However, some limitations remain in the study. First, the aim of the study was to evaluate the overall effect of the dietary supplement on cognitive impairment in aged rats, so we only investigated the effect of integral dietary supplement. The identification of the specific nutrient which counteracts cognitive impairment is beyond the scope of this study, while it remains unknown whether there is a synergistic or antagonistic effect. For instance, a combination of neuroprotective compounds may have synergistic actions that promote neuroimmune protection, reduce the effects of aging, and improve the absorption, bioavailability and actions of neuroprotective compounds in the brain (28). Many nutrients (such as folate, vitamin B_12_, vitamin A, iron and zinc) are found to be capable of regulating n-3 PUFA synthesis (65). Second, as we didn’t aim to observe the effect of the dietary supplement on micronutrient levels in rats, we did not examine the concentrations of the nutrients in blood and tissues. According to previous literature, dietary supplements containing micronutrients and n-3 PUFA can be well absorbed by rats when mixed into feed (15, 66). The concentrations of the nutrients in blood will subsequently be assessed in population-based intervention studies. Moreover, the MWM test is an approach sufficient for assessing cognitive function on its own (67), so we only used the MWM test to assess cognitive function. Future studies using more cognitive assessments are needed to further validate the effectiveness of cognitive enhancement of this dietary supplement.

In summary, we observed that this dietary supplement restored D-gal-induced spatial and memory impairment, accompanied by the up-regulation of BDNF, and the enhancement of antioxidant and anti-neuroinflammatory abilities. Our results provide evidence for further clinical interventions; however, the optimization of a supplement approach in humans requires further clinical trials. Nonetheless, these findings suggest that this dietary supplement could be a promising candidate for the neuroenhancement of age-related cognitive impairments.

## Data Availability Statement

The original contributions presented in the study are included in the article/Supplementary Material, further inquiries can be directed to the corresponding author/s.

## Ethics Statement

The animal study was reviewed and approved by the Animal Ethics Committee of Fudan University (approval number 201810002Z).

## Author Contributions

JS and QR contributed to conceptualization, methodology, supervision, and writing—original draft preparation, review and editing. QR, HX, MY, DX and YZ performed the experiments. QR and DX analyzed the data. All authors have read and agreed to the published version of the manuscript.

## Conflict of Interest

The authors declare that the research was conducted in the absence of any commercial or financial relationships that could be construed as a potential conflict of interest.

## Publisher’s Note

All claims expressed in this article are solely those of the authors and do not necessarily represent those of their affiliated organizations, or those of the publisher, the editors and the reviewers. Any product that may be evaluated in this article, or claim that may be made by its manufacturer, is not guaranteed or endorsed by the publisher.

## Abbreviations

BCA: bicinchoninic acid
BDNF: brain-derived neurotrophic factors
BSA: bovine serum albumin
DG: dentate gyrus
D-gal: D-galactose
DHA: docosahexaenoic acid
DRIs: dietary reference intakes
FDA: Food and Drug Administration
GFAP: glial fibrillary acidic protein
H&E: hematoxylin-eosin
IL-6: interleukin-6
MDA: malondialdehyde
MWM: Morris water maze
n-3 PUFA: n-3 polyunsaturated fatty acids
NSCs/NPCs: neural stem cells and progenitor cells
PBS: phosphate-buffered saline
PS: phosphatidylserine
ROS: reactive oxygen species
SEM: standard error of the mean
SOD: superoxidase dismutase
TNF-α: tumor necrosis factor-α.
