# Supplementary material for: A Dietary Supplement Containing Micronutrients, Phosphatidylserine, and Docosahexaenoic Acid Counteracts Cognitive Impairment in D-Galactose-Induced Aged Rats
Source: Front Nutr. 2022 Jul 5;9:931734. doi: 10.3389/fnut.2022.931734 (PMC9294405; doi:10.3389/fnut.2022.931734)
Supplement: Supplementary file 1 [file Presentation_1.pdf]

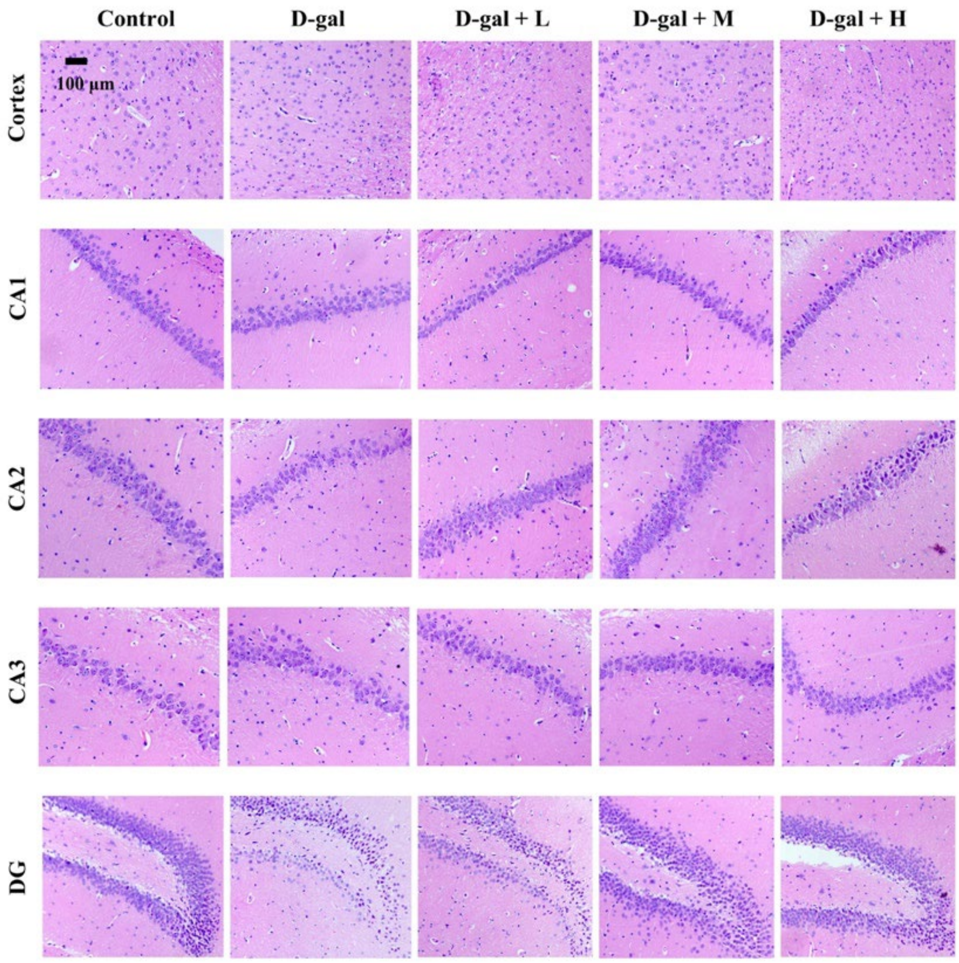

Supplementary Figure 1. The representative photomicrographs of H&E staining in the cortex, CA1-3, and DG region of the hippocampus.

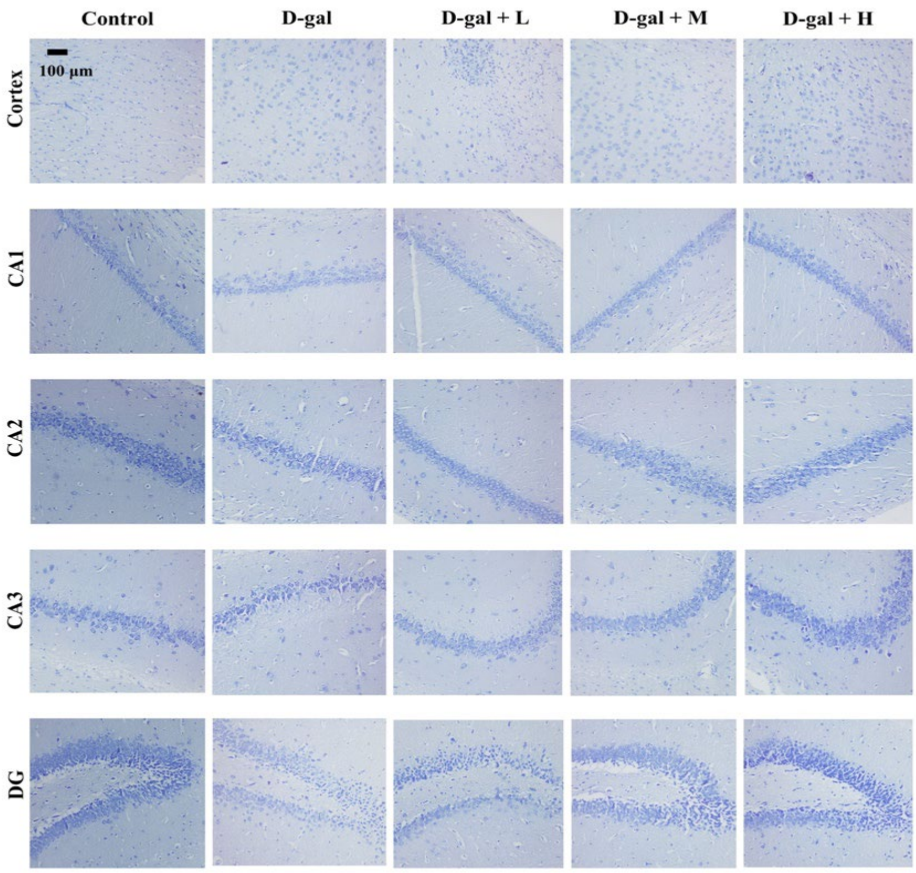

Supplementary Figure 2. The representative photomicrographs of Nissl staining in the cortex, CA1-3, and DG region of the hippocampus.

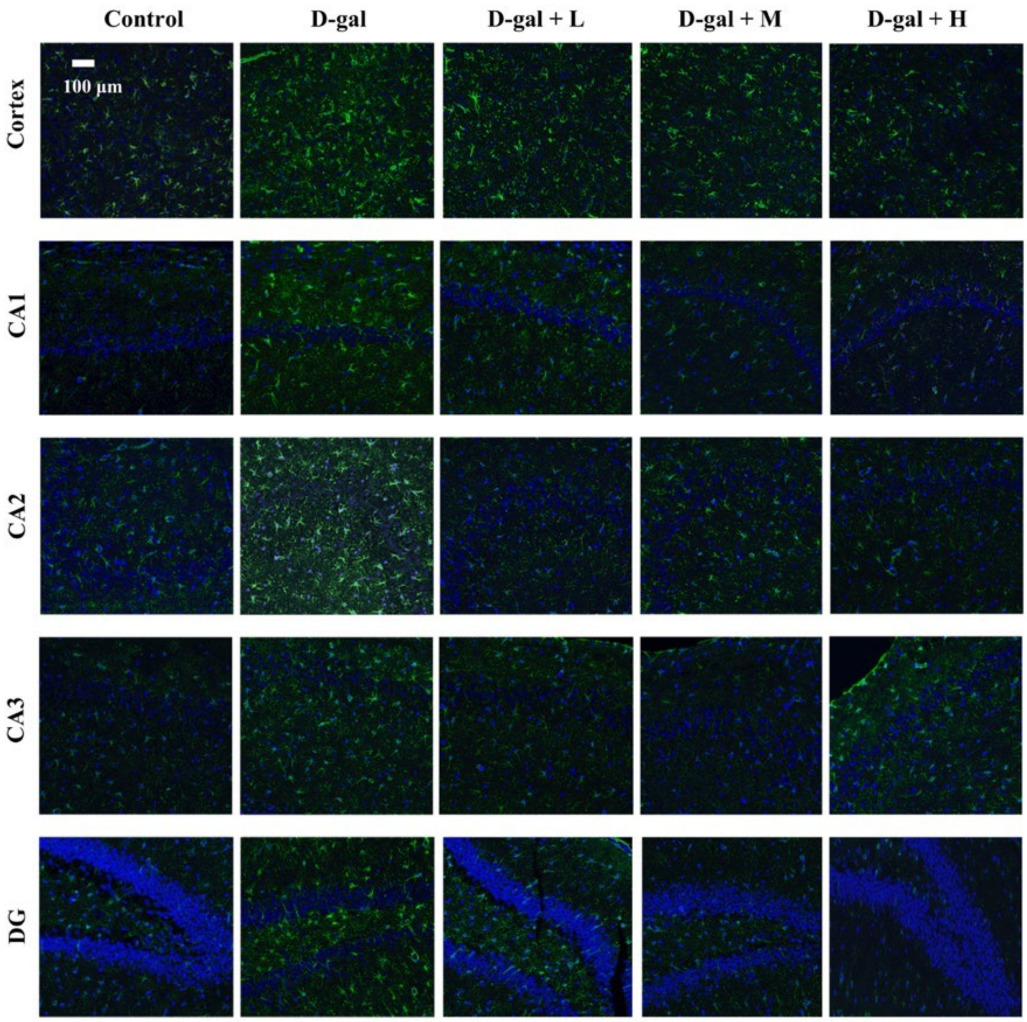

Supplementary Figure 3. The representative photomicrographs of immunofluorescence staining in the cortex, CA1-3, and DG region of the hippocampus.
